# Analyses of human papillomavirus, *Chlamydia trachomatis*, *Ureaplasma urealyticum*, *Neisseria gonorrhoeae*, and co-infections in a gynecology outpatient clinic in Haikou area, China

**DOI:** 10.1186/s12905-023-02259-6

**Published:** 2023-03-21

**Authors:** Zhe Lu, Peizhen Zhao, Huijun Lu, Meifang Xiao

**Affiliations:** Clinical Laboratory, Women and Children’s Health Care Center of Hainan, 75 Longkun Nan Road, Haikou City, 570100 Hainan Province China

**Keywords:** *Chlamydia trachomatis*, *Ureaplasmaurealyticum*, *Neisseria gonorrhoeae*, Human papillomavirus, Reproductive health

## Abstract

**Background:**

The purpose of this study was to study the infection rates of *Chlamydia trachomatis* (CT), *Ureaplasma urealyticum* (UU), *Neisseria gonorrhoeae* (NG), and co-infections with human papillomavirus (HPV) in a hospital gynecology outpatient clinic in the Haikou region in 2021.

**Methods:**

From January to December 2021, the Women and Children Medical Center of Hainan Province collected 2389 samples of cervical exfoliated cells and vaginal swab specimens from gynecologic outpatients. The samples were then analyzed descriptively for data, and the detection rate of each pathogen was tallied. All vaginal swabs were obtained for CT, UU, and NG DNA testing, and cervical exfoliated cells for HPV genotyping. Analyses were performed on the detection rate of each group.

**Results:**

In 2389 samples, the frequencies of pathogen identification among the 2389 samples were as follows: UU (58.43%); HPV (17.29%); CT (7.99%); and NG (0.38%). HPV, CT, UU, and NG were detected in 33.33%, 22.55%, 77.45%, and 2.94% of individuals between 15 and 20 years of age, respectively. The detection rates of CT, UU, and NG were substantially greater in the HPV-positive group than the the HPV-negative group (*P* < 0.05).

**Conclusion:**

Among gynecologic outpatients at a hospital in the Haikou area, the probability of mixed infections with genital tract pathogens in HPV-positive patients was higher compared to HPV-negative patients. Reproductive tract infections are becoming more prevalent in younger people, hence adolescent sexual health education needs improvement.

## Background

Sexually transmitted infections (STIs) are a major source of morbidity globally in both men and women. The use of Pap smears and human papillomavirus (HPV) testing has led to an increase in the detection of cervical dysplasia and a decrease in the incidence of cervical cancer. Mobile health programs enhance cancer screening rates in the general population and are of enormous value in poor nations. New screening technologies, such as p16/Ki67, HPV self-testing, and the application of artificial intelligence in colposcopy evaluations, should be widely used. Focusing on pre-cancerous lesions and developing tools for women at risk for persistent and recurrent pre-cancerous lesions following primary conization (also containing cryo, LEEP), and identifying groups at high risk have clinical value [1–3].

HPV infections are among the most common STIs, and chronic infections with high-risk HPV (hrHPV) genotypes can lead to cervical dysplasia and invasive cervical cancer [4]. Common STI pathogens include HPV, *Chlamydia trachomatis* (CT), *Ureaplasma urealyticum* (UU), and *Neisseria gonorrhoeae* (NG). HPV infections are linked to vaginal microenvironmental dysfunction. Compared to women without bacterial vaginosis (BV), the hrHPV infection rate in women with BV is increased [5]. Furthermore, BV is associated with chronic cervical inflammation that injures the mucosal barrier and compromises immune protection to promote hrHPV infection [6]. For example, condyloma acuminata and cervical lesions are caused by chronic HPV infection, and hrHPV has a significant role in the development of cervical cancer [7]. HPV is known to be the leading cause of cervical cancer (CC), accounting for approximately 311,000 deaths globally in 2018 [8]. Moreover, a small percentage of women with HPV will develop a pre-cancerous or malignant lesion [4]. Although no statistically significant link was demonstrated between hrHPV and other STI co-infections and positive colposcopy findings in the study population, the high prevalence of STIs in hrHPV-positive women, who are at higher risk of developing the cervical disease, suggests that screening for genital infections in this population may be important [4].

CT, UU, and NG infections can result in symptoms, including urethritis and vaginal itching, as well as reproductive health issues, including infertility or miscarriage. NG, UU, and CT infections increase the risk of HPV-mediated cervical malignancies [4, 8, 9], thus early detection of genital tract pathogens is essential for cervical cancer prevention and early cervical cancer screening.

To determine the infection rates of CT, UU, and NG, and co-infections with HPV in the Haikou area of China, we analyzed the overall detection of HPV, CT, UU, and NG and the age distribution of patients in the Gynecology Outpatient Clinic of Hainan Women and Children Medical Center between January and December 2021.

## Data and methods

### Study data

From January to December 2021, a total of 2389 consecutive female patients (mean age, 31.21 ± 4.24 years) who attended our Gynecology Outpatient Clinic with a suspected genital tract infection were chosen. The inclusion criteria were as follows: 1. at least one symptom of vaginitis (abnormal vaginal discharge; painful or frequent urination; vaginal itching, burning, or irritation; painful or uncomfortable intercourse; and vaginal odor); 2. sexual abstinence for ≥ 24 h; 3. No vaginal douching and no medications for ≥ 72 h; 4. no current menses; and 5. signed informed consent. The exclusion criteria were as follows: atypical genital bleeding; use of medications; and unwillingness to participate in the study.

### Sample collection

Vaginal swabs were obtained from all patients and tested for CT, UU, and NG DNA, as well as cervical exfoliated cells for HPV genotyping. The sampling procedures were carried out in accordance with the sampling tube instructions (National Instrument Note, 20,153,401,995). The sample delivery and preservation methods were standardized. The purpose of the study was explained to eligible patients, who gave informed consent before authorization by the Medical Ethics Committee of the Hainan Women and Children Medical Center (HNWCMC2021-40).

### Research methods

#### Nucleic acid extraction

Nucleic acids were extracted from samples using a magnetic bead technique kit from Shenzhen Ruineng Biotechnology Co., Ltd. (Shenzhen, China), and the procedure was followed precisely according to the kit's instructions (National Instruments Note, 20,143,801,491).

#### HPV detection

The sample HPV typing test was performed using a 21 HPV GenoArray Diagnostic Kit (HBGA-21PKG; Hybribio, Kowloon Bay, Hong Kong, China), which detects HPV 6, 11, 16, 18, 31, 33, 35, 39, 42, 43, 44 45, 51, 52, 53, 56, 58, 59, 66, 68, and cp8304. All steps were performed according to the manufacturer’s instructions. The detection instrument was a Hema-9600 [10] with fully automated nucleic acid molecular hybridization technology (HBHM-9001A; Hybribio).

#### CT, UU, and NG assays

The *Chlamydia trachomatis* Diagnostic Kit (DA 0071), *Ureaplasma urealyticum* Diagnostic Kit [DA1120], and *Neisseria gonorrhoeae* Test Kit [DA0063] (all from The DaAn Gene Co., Ltd., Guangdong, China) were used to identify the three pathogens, and all procedures were carried out in accordance with the instructions on the kit packaging. The SLAN-96P real-time fluorescence quantitative PCR apparatus was used for DNA detection (Shanghai Toujing Life Technology Co., Shanghai, China).

### Statistical methods

SPSS 26.0 software was used for statistical analyses. Count data are expressed as n(%). A χ2 or Fisher exact test was used to compare rates between groups, as indicated. The difference was considered statistically significant at a *P* < 0.05.

## Results

### Detection of HPV, CT, UU, and NG in 2389 patient samples

Among the samples tested, UU had the highest detection rate (56.84% [1358/2389]), followed by HPV (17.29% [413/2389]), CT (7.99% [191/2389]), and NG (0.38% [9/2389]).

### Detection of different HPV subtypes

A total of 413 HPV-positive samples were detected from 2398 patients. The total HPV subtype detection rate was 16.71% (69/413); HPV subtype 52 had the highest detection rate. All 21 HPV subtypes detectable in the kit were detected. The detection rates for the top 5 HPV subtypes were as follows: 52 (69/413); 16 (56/413); 39 (38/413); 51 (38/413); and 58 (36/413).

### Detection of pathogens in different age groups (Table 1)

**Table 1:** Detection of pathogens in different age groups [n(%)]

Age (year)	n	HPV	CT	UU	NG
		Positive cases	Positive cases	Positive cases	Positive cases
15 ~ 20	102	34 (33.33%)^a^	23(22.55%)^a^	79(77.45%)^a^	3(2.94%)^a^
21 ~ 25	547	114(20.84%)^b^	72(13.16%)^b^	345(63.07%)^b^	4(0.73%)^b^
26 ~ 30	816	125(15.32%)	42(5.15%)	447(54.78%)	1(0.12%)
31 ~ 35	505	84(16.63%)	44(8.71%)	249(49.31%)	0(0.00%)
36 ~ 40	241	36(14.94%)	8(3.32%)	136(56.43%)	0(0.00%)
> 41 ~	178	20(11.24%)	2(1.12%)	102(57.30%)	1(0.56%)
Total	2389	413(17.29)	191(7.99%)	1358(56.84%)	9(0.38%)

*HPV* Human papillomavirus, *CT Chlamydia trachomatis*, *UU Ureaplasma urealyticum*, *NG Neisseria gonorrhoeae*

a: *P* < 0.05 when compared with groups 21–25 (*P* = 0.013), 26–30 (*P* = 0.008), 31–35 (*P* = 0.008), 36–40 (*P* = 0.008), and ≥ 41 (*P* = 0.005); b: *P* < 0.05 when compared with groups 26–30 (*P* = 0.022), 31–35 (*P* = 0.025), 36–40 (*P* = 0.022), and ≥ 41 (*P* = 0.017)

The results showed that the HPV, CT, UU, and NG detection rates were the highest in the 15–20 year age group; the difference was statistically significant compared with the other age groups (*P* < 0.05). The HPV, CT, UU, and NG detection rates in the 21–25 year age group was second only to the 15–20 year age group; the difference was statistically significant compared with the other age groups (*P* < 0.05). There were no significant differences in the HPV, CT, UU, and NG detection rates between the 26–30, 31–35, 36–40, and ≥ 41 year age groups.

### Mixed HPV infections with CT, UU, and NG (Table 2)

**Table 2:** Co-infection of HPV with CT, UU, and NG [n(%)]

Groups	CT	UU	NG
	Positive cases	Positive cases	Positive cases
HPV-positive (*n* = 413)	57(13.80%)	323(78.21%)	6(1.45%)
HPV-negative (*n* = 1976)	134(6.78%)	1035(52.38%)	3(0.15%)
Total (*n* = 2389)	191(7.99%)	1358(56.84%)	9(0.38%)
χ^2^ value	22.886	92.904	15.405
*P* value	< 0.001	< 0.001	< 0.001

*CT Chlamydia trachomatis*, *UU Ureaplasma urealyticum*, *NG Neisseria gonorrhoeae*, *HPV* Human papillomavirus

The samples were separated into groups of HPV-positive and -negative patients based on the results of HPV tests. CT, UU, and NG detection in each group was recorded. CT [(57 (13.80%) vs. 134 (6.78%); *P* < 0.001], UU [323 (78.21%) vs. 1035 (52.38%); *P* < 0.001], and NG [6 (1.45%) vs. 3 (0.15%); *P* < 0.001] detection rates were significantly higher in the HPV-positive group than the HPV-negative group.

## Discussion

The HPV detection rate in outpatients was 17.29%, which is higher than the average rate of HPV carriage among Chinese women countrywide (11.2%) [11]. The top 5 HPV subtypes found in Haikou were 52, 16, 39, 51, and 58, which were different from the top HPV subtypes in Shanghai and Yunnan, according to the data currently available, which suggests that geographic location may have an impact on the HPV subtype detection rate [12, 13].

The findings of this study demonstrated that among patients with an HPV infection, there was a higher likelihood of detecting CT, UU, and NG in cervical samples. The pathogen identification frequencies among the 2389 samples were as follows: UU (58.43%); HPV (17.29%); CT (7.99%); and NG (0.38%). The prevalence of UU has been reported to be 20% in South America, 41.9% in Italy, and 51.5% in Africa [14]. The highest prevalence of UU (53.8%) was detected in females between 34 and 39 years of age [15].

HPV is mostly spread via sexual contact, and the likelihood of infection increases with age and sexual behavior [16]. HPV is important, but not sufficient for cervical carcinogenesis, suggesting the involvement of other variables. The vaginal microbiota is a key factor in managing HPV infections, and depending on the composition, the vaginal microbiota can alter the vaginal mucosa microenvironment against viral infections. Numerous studies have shown an increased risk of HPV infection at younger ages [17–19]. Specifically, the highest prevalence of HPV occurs among adolescents and young adults between the ages of 15 and 25 years. It has been estimated that > 75% of new HPV infections occur in individuals in this age range [17–19]. This increased risk of infection in younger women has been linked to a lack of adaptive immune responses and/or a relatively large area of cervical epithelium undergoing squamous metaplasia in this age group, which may increase the opportunity for HPV DNA to infect the basal cell layer, then proliferate [20]. In agreement with local and international studies [21], we showed that the 15–25 year age range in gynecologic outpatients has a higher HPV detection rate than the total HPV detection rate.

The primary pathogens that cause infections of the female reproductive system are HPV, CT, UU, and NG. In the early stages of infection, these pathogens frequently cause no or moderate symptoms, which makes it easy to miss the diagnosis or misdiagnose the symptoms and cause recurring sickness, such as non-gonococcal urethritis, pelvic inflammation, gonorrhea, and chronic cervicitis [21, 22]. In contrast, mixed infections caused by several different microorganisms make it difficult to diagnose and treat the illness.

The probability of HPV infection is increased by polymicrobial co-infections in addition to age-related variables [23]. Cervical mucosa, which acts as a protective barrier against pathogens penetrating the upper female reproductive system, is frequently challenged by pathogens and dysbiosis [24]. Co-infections are becoming more common in clinical practice, although the role in disease progression is unknown [25]. Co-infections with CT are more common in patients with invasive cervical and ovarian malignancies [26]. The impact of co-existing HPV and CT in a stem cell could be detrimental to cellular and genomic integrity, thus promoting neoplastic growth. CT and HPV generate unique transcriptional and post-translational responses, resulting in distinct reprogramming of host cell processes. Surprisingly, CT interferes with HPV-induced mechanisms that maintain cellular and genomic integrity, such as mismatch repair in stem cells [25].

CT is recognized as a significant co-factor for HPV infections [27]. Other STIs are increasingly being implicated as co-factors in the development of cervical cancer in HPV-positive women [28]. Interactions between HPV and other infections with comparable mucosal locations may hasten cancer progression by increasing HPV replication and infection persistence. Recent studies, for example, have reported an elevated risk of cervical cancer in women infected with HPV and CT [29]. Both HPV and CT cause changes in the cervical immune system and vaginal microbial ecosystem. According to Seraceni et al. [30], cervical cancer caused by HPV is strongly correlated with the presence of a CT infection, which makes it easier for HPV to infect cervical epithelial cells. In contrast to an HPV mono-infection, Khan et al. [31] showed that a CT-HPV mixed infection is related to modifications in the oncogenic protein expression profile of cervical cells, which may help increase HPV infection when CT is present.

The pathogen that most often infects the female reproductive system, UU, has recently been identified as one of the major risk factors for the development of HPV infection or cervical dysplasia [32]. When UU and HPV infection are combined, Wang et al. [33] reported an elevated incidence of high-grade squamous intraepithelial lesions plus invasive cervical cancer [33]. Based on a meta-analysis, Ye [32] concluded that UU enhanced the rate of high-risk HPV infections and the progression to cervical cancer. Co-infection with HPV and UU has previously been reported in males with urethritis [34].

Although there were few NG-positive samples and a low incidence of NG infections in our study, we did show a higher rate of NG infections in the HPV-positive group. In addition, we showed a low rate of NG infections and a low number of NG-positive samples, but still found an increased rate of NG infections in the HPV-positive group. This finding suggests that NG infections also increase the risk of HPV infections and should be taken seriously and treated aggressively. Early identification of genital tract pathogens is important for the prevention and treatment of cervical cancer [21, 23], and because infection with CT, UU, and NG enhances HPV-mediated cervical malignant lesions [35], our study showed the practical value of combined HPV, UU, CT, and NG testing.

It is currently thought that genital tract infections are closely associated with age and sexual habits. An age-specific prevalence curve revealed that HPV infections first peak in women < 30 years of age, then decrease with age, but the curve surges again at approximately 50 years of age and peaks again in women ≥ 60 years of age [6]. Due to the lack of sex education, adolescents are more likely to have high-risk sexual behavior and neglect treatment. From a biological perspective, adolescent females are more likely to be infected with reproductive tract pathogens, such as CT and HPV, due to reduced cervical mucus secretion and increased cervical ectopion [36, 37]. According to a study conducted from 2005–2015, the initial age of cervical cancer in Chinese women appears at approximately 25 years of age, then the incidence of cervical cancer tends to decrease [38].

In recent years it has been shown that the time of sexual debut in the adolescent student population (15–24 years of age) in China is earlier and the proportion is increasing, and understanding the need for self-protection is inadequate [39]. A questionnaire survey related to the sexual behavior of adolescents 14–24 years of age in Michigan (USA) pointed out that the lack of family and school education, as well as proper guidance by the media, is the cause of high-risk sexual behavior among adolescents [40]. According to recent research, Blacks are less likely to be infected with HPV16 than Whites, based on self-reported race and genomic ancestry-relevant markers, but are more likely to be positive for other carcinogenic HPV strains [41, 42].

In this study we found a greater incidence of genital tract pathogens in patients between 15 and 25 years of age in the Haikou gynecologic clinics, indicating an uptick in genital tract infections in this age range. It is more important to increase reproductive health knowledge from families, schools, and networks to give early reproductive health education to young women and encourage them adopt prudent reproductive health protective measures. Indeed, this is crucial to lowering reproductive tract infections and enhancing the reproductive health of women who are ready to have children. Small quantities of RNA and DNA, as well as tumor cells, may now be detected in peripheral blood using technological advances, such as circulating tumor cells (CTCs), circulating cell-free DNA (cfDNA), circulating HPV DNA, and miRNA. Circulating molecules and biomarkers have the potential to be a useful diagnostic and prognostic tool for cervical cancer [43]. Furthermore, the presence of hrHPV types, positive endocervical margins, HPV persistence, and the omission of HPV vaccination after conization increase the risk of developing cervical dysplasia persistence and/or recurrence independent of the risk of developing cervical dysplasia persistence and/or recurrence. A nomogram is an effective tool for counseling women about their likelihood of cervical lesion recurrence following initial conization. Triaging high-risk patients in specialist facilities and tailoring more suitable follow-up plans may be beneficial [44].

The single hospital from which all of the patients were chosen was a limitation that may have reduced the ability to generalize the findings. Future studies should include subgroup studies on age and co-infections with HPV, CT, UU, and NG.

## Conclusions

Among Haikou gynecologic outpatients, UU is the genital tract infection with the highest detection rate. To prevent mixed infections from genital tract pathogens, integrated polymicrobial testing is warranted in HPV-positive carriers. Reproductive tract infections are on the rise in younger people, thus adolescent sexual health education must be improved.

## Data Availability

All data analyzed during this study are included in this published article.

## Abbreviations

HPV: Human papillomavirus
CT: *Chlamydia trachomatis*
UU: *Ureaplasma* *urealyticum*
NG: *Neisseria gonorrhoeae*
STIs: Sexually transmitted infections

## References

[1] Bogani G, Sopracordevole F, Di Donato V, Ciavattini A, Ghelardi A, Lopez S (2021). High-Risk HPV-positive and -negative high-grade cervical dysplasia: analysis of 5-year outcomes. Gynecol Oncol.

[2] Poniewierza P, Panek G (2022). Cervical cancer prophylaxis-state-of-the-art and perspectives. Healthcare (Basel).

[3] Giannini A, Bogani G, Vizza E, Chiantera V, Laganà AS, Muzii L (2022). Advances on prevention and screening of gynecologic tumors: are we stepping forward?. Healthcare (Basel).

[4] Martinelli M, Musumeci R, Sechi I, Sotgiu G, Piana A, Perdoni F (2019). Prevalence of human papillomavirus (HPV) and other sexually transmitted infections (STIs) among Italian women referred for a colposcopy. Int J Environ Res Public Health.

[5] Lin W, Zhang Q, Chen Y, Dong B, Xue H, Lei H (2022). Changes of the vaginal microbiota in HPV infection and cervical intraepithelial neoplasia: a cross-sectional analysis. Sci Rep.

[6] Lv P, Zhao F, Xu X, Xu J, Wang Q, Zhao Z (2019). Correlation between common lower genital tract microbes and high-risk human papillomavirus infection. Can J Infect Dis Med Microbiol.

[7] Hu SY, Rezhake R, Chen F, Zhang X, Pan QJ, Ma JF (2020). Outcomes in women with biopsy-confirmed cervical intraepithelial neoplasia grade 1 or normal cervix and related co-factors: A 15-year population-based cohort study from China. Gynecol Oncol.

[8] Ferlay J, Ervik M, Lam F, Colombet M, Mery L, Piñeros M, et al. Global Cancer Observatory: Cancer Today. International Agency for Research on Cancer; Lyon, France: 2018. https://gco.iarc.fr/today/home.

[9] Jary A, Teguete I, Sidibé Y, Kodio A, Dolo O, Burrel S (2021). Prevalence of cervical HPV infection, sexually transmitted infections and associated antimicrobial resistance in women attending cervical cancer screening in Mali. Int J Infect Dis.

[10] http://www.kyangou.com/p-V512818.html.

[11] Zhao FH, Lewkowitz AK, Hu SY, Chen F, Li LY, Zhang QM (2012). Prevalence of human papillomavirus and cervical intraepithelial neoplasia in China: a pooled analysis of 17 population-based studies. Int J Cancer.

[12] Cao JX, Li XF, Shen GL, Chu Q (2021). Survey of HPV subtype infection in women in seven regions of Yunnan. Lab Med Clin.

[13] Xuan BB, Tan MY, Sun HX, Sheng HM (2020). Analysis of mixed infections of human papillomavirus with Ureaplasma urealyticum, Chlamydia trachomatis and Neisseria gonorrhoeae in changning district. Shanghai Laboratory Medicine.

[14] Leli C, Mencacci A, Bombaci JC, D'Alo F, Farinelli S, Vitali M (2012). Prevalence and antimicrobial susceptibility of Ureaplasma urealyticum and Mycoplasma hominis in a population of Italian and immigrant outpatients. Infez Med.

[15] Maleki S, Motamedi H, Moosavian SM, Shahbaziyan N (2013). Frequency of mycoplasma hominis and ureaplasma urealyticum in females with urogenital infections and habitual abortion history in ahvaz, iran; using multiplex PCR. Jundishapur J Microbiol.

[16] Zhao FH, Tiggelaar SM, Hu SY, Zhao N, Hong Y, Niyazi M (2012). A multi-center survey of HPV knowledge and attitudes toward HPV vaccination among women, government officials, and medical personnel in China. Asian Pac J Cancer Prev.

[17] Dunne EF, Unger ER, Sternberg M, McQuillan G, Swan DC, Patel SS (2007). Prevalence of HPV infection among females in the United States. JAMA.

[18] Kahn JA, Lan D, Kahn RS (2007). Sociodemographic factors associated with high-risk human papillomavirus infection. Obstet Gynecol.

[19] Manhart LE, Holmes KK, Koutsky LA, Wood TR, Kenney DL, Feng Q (2006). Human papillomavirus infection among sexually active young women in the United States: implications for developing a vaccination strategy. Sex Transm Dis.

[20] Trottier H, Franco EL (2006). Human papillomavirus and cervical cancer: burden of illness and basis for prevention. Am J Manag Care.

[21] Liu QZ (2014). Guidelines for the treatment of condyloma acuminatum (2014). Chin J Dermatol.

[22] Center for STD Control, Chinese Center for Disease Control and Prevention- Venereal Diseases Group, Chinese Society of Medical Sciences, Division of Dermatology and Venereology Subspecialty Committee of Venereal Diseases, Dermatologists Branch, Chinese Medical Association (2020). Guidelines for the diagnosis and treatment of syphilis, gonorrhea and genital tract Chlamydia trachomatis infection (2022). Chin J Dermatology.

[23] Zhang HY, Tiggelaar SM, Sahasrabuddhe VV, Smith JS, Jiang CQ, Mei RB (2012). HPV prevalence and cervical intraepithelial neoplasia among HIV-infected women in Yunnan Province, China: a pilot study. Asian Pac J Cancer Prev.

[24] Lacroix G, Gouyer V, Gottrand F, Desseyn JL (2020). The Cervicovaginal Mucus Barrier. Int J Mol Sci.

[25] Koster S, Gurumurthy RK, Kumar N, Prakash PG, Dhanraj J, Bayer S (2022). Modelling Chlamydia and HPV co-infection in patient-derived ectocervix organoids reveals distinct cellular reprogramming. Nat Commun.

[26] Koskela P, Anttila T, Bjørge T, Brunsvig A, Dillner J, Hakama M (2000). Chlamydia trachomatis infection as a risk factor for invasive cervical cancer. Int J Cancer.

[27] Silva J, Cerqueira F, Medeiros R (2014). Chlamydia trachomatis infection: implications for HPV status and cervical cancer. Arch Gynecol Obstet.

[28] Wang L, Zhu L, Li H, Ma N, Huang H, Zhang X (2019). Association between asymptomatic sexually tranmitted infections and high-risk human papillomavirus in cervical lesions. J Int Med Res.

[29] Mancini F, Vescio F, Mochi S, Accardi L, di Bonito P, Ciervo A (2018). HPV and Chlamydia trachomatis coinfection in women with Pap smear abnormality: Baseline data of the HPV Pathogen ISS study. Infez Med.

[30] Seraceni S, De Seta F, Colli C, Del Savio R, Pesel G, Zanin V (2014). High prevalence of hpv multiple genotypes in women with persistent chlamydia trachomatis infection. Infect Agents Cancer.

[31] Khan AA, AAbuderman A, Ashraf MT, Khan Z (2020). Protein-protein interactions of HPV-Chlamydia trachomatis-human and their potential in cervical cancer. Future Microbiol.

[32] Ye H, Song T, Zeng X, Li L, Hou M, Xi M (2018). Association between genital mycoplasmas infection and human papillomavirus infection, abnormal cervical cytopathology, and cervical cancer: a systematic review and meta-analysis. Arch Gynecol Obstet.

[33] Wang L, Cai H, Yang X, Huang HF, Fang J (2020). Correlation between occult infection with sexually transmitted pathogens and high-risk HPV and cervical lesions. J Xi'an Jiaotong Univ (Medical Edition).

[34] Shigehara K, Kawaguchi S, Sasagawa T, Furubayashi K, Shimamura M, Maeda Y (2011). Prevalence of genital Mycoplasma, Ureaplasma, Gardnerella, and human papillomavirus in Japanese men with urethritis, and risk factors for detection of urethral human papillomavirus infection. J Infect Chemother.

[35] Liang Y, Chen M, Qin L, Wan B, Wang H (2019). A meta-analysis of the relationship between vaginal microecology, human papillomavirus infection and cervical intraepithelial neoplasia. Infect Agent Cancer.

[36] Shannon CL, Klausner JD (2018). The growing epidemic of sexually transmitted infections in adolescents: a neglected population. Curr Opin Pediatr.

[37] https://www.cdc.gov/std/stats16/CDC_2016_STDS_Report-for508WebSep21_2017_1644.pdf.

[38] Li X, Liu C, Zhou W, He MY, Xiong WJ, Rang WQ (2021). Trend analysis of cervical cancer incidence and mortality in China from 2005 to 2015. J Huazhong Univ Scien and Techn: Medical Edition.

[39] Han J, Mao YR, Tang HL, Li J, Wu ZY (2018). Analysis of first follow-up and CD4+ T-lymphocyte testing among newly reported young students with HIV infection in China, 2013–2017. Chin J Prev Med.

[40] Strome A, Moore-Petinak N, Waselewski M, Chang T (2022). Youths' knowledge and perceptions of health risks associated with unprotected oral sex. Ann Fam Med.

[41] Keller MJ, Burk RD, Massad LS, Eltoum IE, Hessol NA, Anastos K (2018). Racial differences in human papilloma virus types amongst United States women with HIV and cervical precancer. AIDS.

[42] Montealegre JR, Peckham-Gregory EC, Marquez-Do D, Dillon L, Guillaud M, Adler-Storthz K (2018). Racial/ethnic differences in HPV 16/18 genotypes and integration status among women with a history of cytological abnormalities. Gynecol Oncol.

[43] D'Oria O, Corrado G, Laganà AS, Chiantera V, Vizza E, Giannini A (2022). New Advances in Cervical Cancer: From Bench to Bedside. Int J Environ Res Public Health.

[44] Bogani G, Lalli L, Sopracordevole F, Ciavattini A, Ghelardi A, Simoncini T (2022). Development of a nomogram predicting the risk of persistence/recurrence of cervical dysplasia. Vaccines (Basel).

